# Developmental biology of *Spiralicellula* and the Ediacaran origin of crown metazoans

**DOI:** 10.1098/rspb.2024.0101

**Published:** 2024-05-29

**Authors:** Weichen Sun, Zongjun Yin, Pengju Liu, Maoyan Zhu, Philip Donoghue

**Affiliations:** ^1^ State Key Laboratory of Palaeobiology and Stratigraphy, Nanjing Institute of Geology and Palaeontology, Chinese Academy of Sciences, Nanjing 210008, People's Republic of China; ^2^ Nanjing College, University of Chinese Academy of Sciences, Nanjing 211135, People's Republic of China; ^3^ Institute of Geology, Chinese Academy of Geological Sciences, Beijing 100037, People's Republic of China; ^4^ Bristol Palaeobiology Group, School of Earth Sciences, University of Bristol, Life Sciences Building, Tyndall Avenue, Bristol BS8 1TQ, UK

**Keywords:** ediacaran, doushantuo formation, weng'an biota, origin of crown metazoans

## Abstract

The early Ediacaran Weng'an biota (Doushantuo Formation, South China) provides a rare window onto the period of Earth history in which molecular timescales have inferred the initial phase of crown-metazoan diversification. Interpretation of the embryo-like fossils that dominate the biota remains contentious because they are morphologically simple and so difficult to constrain phylogenetically. *Spiralicellula* from the Weng'an biota is distinguished by spiral internal bodies, allied through development to *Megasphaera* or *Helicoforamina* and interpreted variously as metazoan embryos, encysting protists, or chlorophycean green algae. Here we show, using X-ray microtomography, that *Spiralicellula* has a single-layered outer envelope and no more than 32 internal cells, often preserving a nucleus and yolk granules. There is no correlation between the extent of spiral development and the number of component cells; rather, the spiral developed with each palintomic stage, associated with cell disaggregation and reorientation. Evidence for envelope thinning and cell loss was observed in all developmental stages, reflecting non-deterministic shedding of gametes or amoebae. The developmental biology of *Spiralicellula* is similar to *Megasphaera* and *Helicoforamina*, which otherwise exhibit more rounds of palintomy. We reject a crown-metazoan affinity for *Spiralicellula* and all other components of the Weng'an biota, diminishing the probability of crown-metazoan diversification before the early Ediacaran.

## 1. Introduction

**Figure 1.**
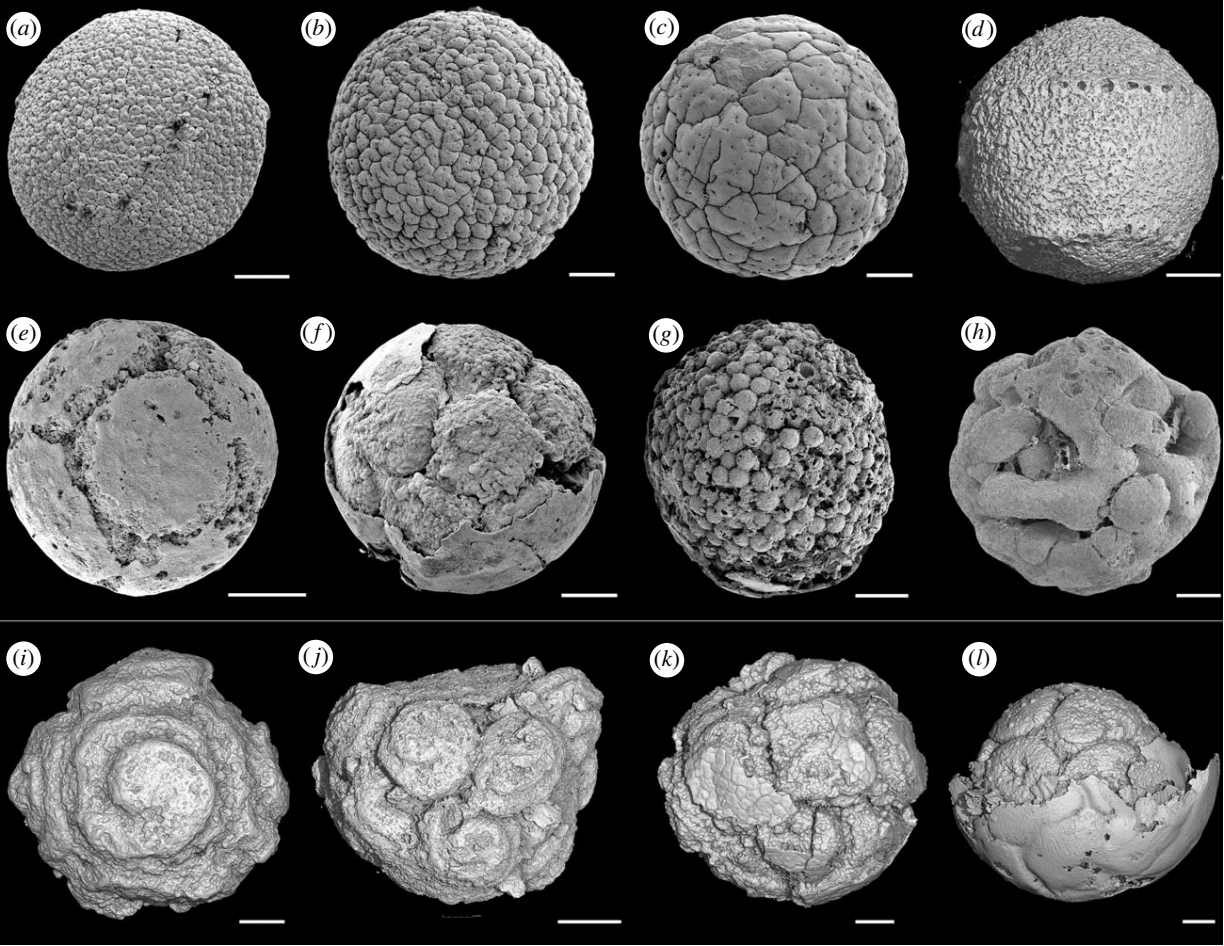
Different types of embryo-like fossils from the Weng'an biota. (*a*–*c*) Scanning electron microscopy (SEM) images of *Megasphaera*. (*d*) Synchrotron radiation X-ray tomographic microscopy (srXTM) surface rendering image of *Helicoforamina*. (*e*,*f*) SEM images of *Parapandorina*. (*g*) SEM image of *Megaclonophycus*. (*h*) SEM image of *Caveasphaera*. (*i–l*) srXTM surface rendering images of *Spiralicellula*. (*a*) B002-105z_0020, (*b*) B002-79z_0041, (*c*) B002-63z_0025, (*d*) ZY-LXB1-B07, (*e*) B003-119z_0047, (*f*) B002-84z_0017, (*g*) B002-104z_0001, (*h*) B005-193z_0038, (*i*) PD2002-76, (*j*) PD2108-02, (*k*) PD2108-24, (*l*) PD2108-17. Scale bars: (*a*,*d*,*f*,*i*) 150 µm; (*b*,*c*,*g*)120 µm; (*e*,*k*,*l*) 100 µm; (*h*) 90 µm; (*j*) 110 µm.

Molecular clock analyses estimate metazoans to have originated before the Cryogenian and diversified in the Ediacaran or Cryogenian [1,2]. These timescales significantly predate the first appearance of unequivocal animals in the middle Ediacaran (approx. 574 Ma) [3], though this may be because early animals lacked cuticle and skeletal mineral, precluding routine fossilization [4], or due to the lack of suitable environments for their fossilization during the Cryogenian period [5]. Hence, the importance of the Weng'an biota (*ca* 590–575 Ma) [6], which provides a window of exceptional fossil preservation onto shallow marine communities in the early-middle Ediacaran [7]. The Weng'an biota is composed largely of microscopic three-dimensional embryo-like fossils [8] preserved to organelle-level fidelity [9]. An animal embryo interpretation has been widely supported [10–19], but the fossils have also been compared to giant bacteria [20], non-metazoan holozoans [21], stem metazoans [9,22] and multicellular chlorophyte [22,23] and streptophyte algae [24]. A majority of the fossils, in terms of abundance, are assigned to the morphotaxon *Megasphaera* (senior synonym of *Parapandorina* and *Megaclonophycus*) but there is a broader diversity including *Yinitianzhushania*, *Caveasphaera*, *Helicoforamina* and *Spiralicellula* (figure 1), which have variably be considered distinct taxa or different components of the life cycle of a smaller number of taxa (e.g. [14,21,25]). *Megaclonophycus* is generally interpreted as a later stage in the life cycle of *Megasphaera*, while *Helicoforamina* and *Spiralicellula* have often been associated as different developmental stages, largely on the basis that they both exhibit helical surface structures.

*Spiralicellula* individuals are composed of geometric clusters of cells that are distinguished by a spiral on the surface of each cell. In establishing *Spiralicellula bulbifera*, Xue *et al.* [26] compared it to the extant volvocalean chlorophyte alga *Pandorina*. Most subsequent studies allied *Spiralicellula* to *Helicoforamina* based on the shared presence of dextrally helical (or spiral) structures [13,14,21,25,27,28], though this has been contested by others [29,30]. Xiao and colleagues inferred palintomic cleavage in *Spiralicellula*, which they interpreted to reflect later development stages of a one-cell *Helicoforamina* of animal affinity [13,14]. Tang *et al*. [27] compared *Spiralicellula* and *Helicoforamina* with adult octocorals and ctenophores, concluding that they represent embryos of the putative fossil ctenophore *Eoandromeda*. Huldtgren *et al*. [21] interpreted the coiled structures in both *Spiralicellula* and *Helicoforamina* as amoeboid cells. Zhang & Pratt [25] reinterpreted *Spiralicellula* and *Helicoforamina* as alternating sexual and asexual life cycles of a chlorophycean alga, comparing the thick fossil envelopes to the cyst walls that enclose zygospores. More recently, Yin *et al*. [30] have revealed that *Helicoforamina* encompasses developmental stages equivalent to those of *Spiralicellula*, suggesting that *Helicoforamina* warrants classification as a distinct taxon, and has been interpreted as a holozoan. However, the precise phylogenetic position of *Helicoforamina* within holozoan phylogenetic tree remains uncertain [30].

*Spiralicellula* is the last key component of the Weng'an biota that has not been fully characterized in terms of its development, key to reconciling green algal [25,26], non-metazoan holozoan [21] and metazoan [13] hypotheses of affinity. Given the potential of *Spiralicellula* to inform on the timing of early animal evolution, we undertook tomographic analysis of a large collection of exceptionally well preserved specimens to characterize the developmental biology of *Spiralicellula*.

## 2. Material and methods

The Weng'an biota occurs in the Doushantuo Formation in the Weng'an phosphate mining area of Guizhou Province, southwest China [7,31]. Stratigraphy and tectonic information of the Doushantuo Formation at Weng'an are described thoroughly elsewhere [7,32]. The studied specimens of *Spiralicellula bulbifera* were collected from the Upper Grey Facies of the Doushantuo Formation (grey phosphorite of unit 4B) at Beidoushan Mine. The phosphatic fossils were released from their calcium carbonate matrix using 7% acetic acid and the ensuing insoluble residue was handpicked with the aid of a stereomicroscope. *Sporosphaera* [33], *Megasphaera* [22], *Helicoforamina* [30] and *Ostiosphaera* [34] co-occur with *Spiralicellula* in these samples.

Well preserved specimens were subjected to microtomography using synchrotron radiation X-ray tomographic microscopy (srXTM) at the TOMCAT (X02DA) beamline of the Swiss Light Source (SLS; Paul Scherrer Institute, Villigen, Switzerland). srXTM data were obtained using 10× and 20× objective lenses (yielding reconstructed tomographic data with voxel dimensions of 0.65 and 0.325 µm, respectively) at energy levels of 18 to 27 keV and exposure times of 180 to 800 ms. In total, 1501 X-ray equiangular projections were obtained as the specimens were rotated through 180° within the beam. Projections were post-processed and rearranged into flat- and dark-field-corrected sinograms, and reconstruction was performed on a 60-core Linux PC farm, using a highly optimized routine based on the Fourier transform method and a regridding procedure [35]. Slice data were analysed and processed using VG StudioMax 3.5 (www.volumegraphics.com). Given that the X-rays from synchrotron sources are monochromatic, differences in contrast in the resulting tomographic slices reflect the densities of the fossil materials through which they pass.

All the specimens figured in this paper have been deposited in NIGPAS. The tomographic data arising from our study are available from the three-dimensional model database of NIGPAS (https://doi.org/10.12091/fossil-ontology.20240104).

## 3. Results

**Figure 2.**
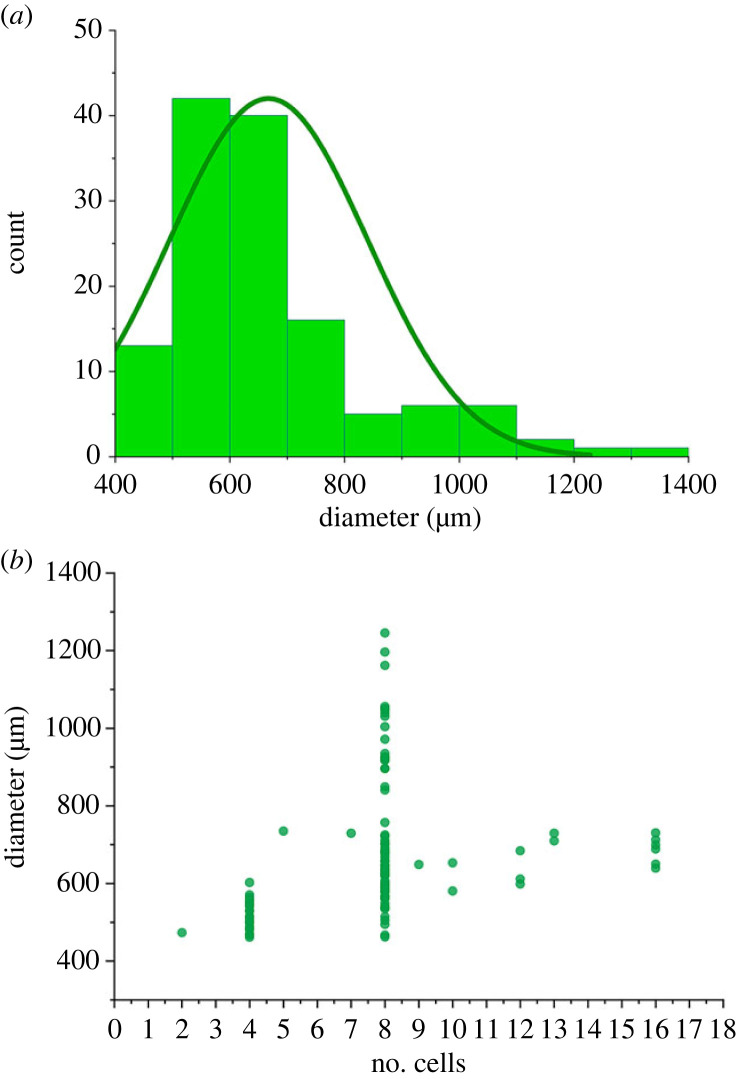
The cell number and diameter of *Spiralicellula*.

**Figure 3.**
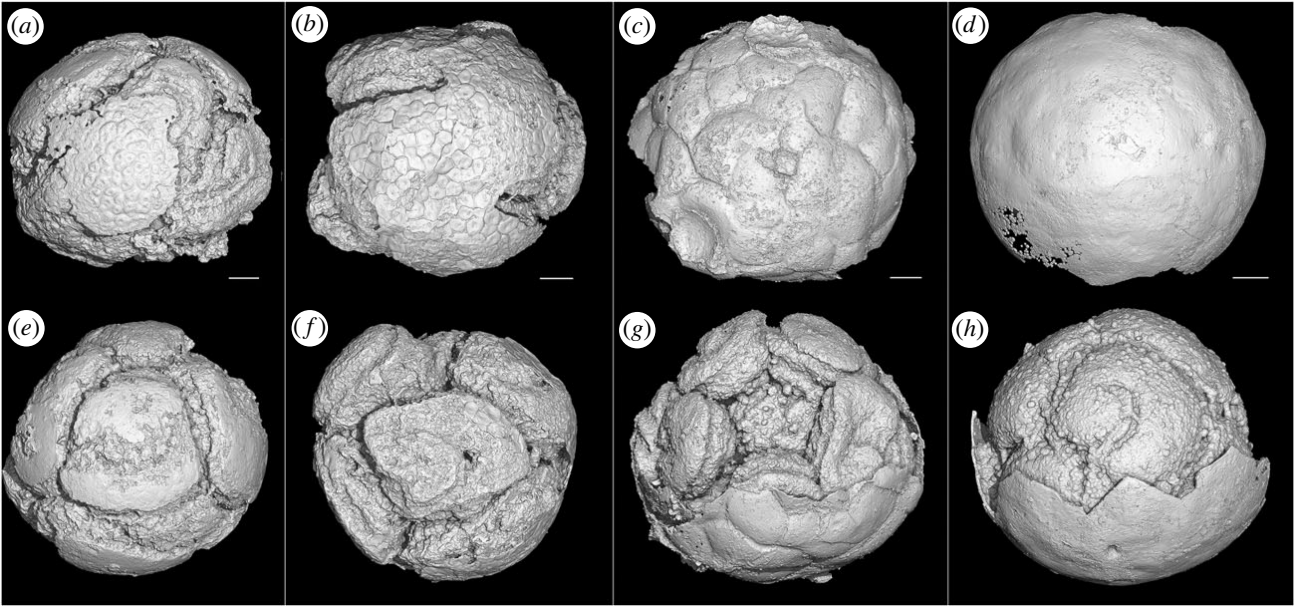
*Spiralicellula* with different types of ornamented envelopes*.* All the images are synchrotron radiation X-ray tomographic microscopy (srXTM) surface renderings showing tumour-like shape (*a*), small polygonal (*b*), large irregular-polygonal (*c*) and smooth (*d*) envelopes. (*a*,*e*) PD2108-14, (*b*,*f*) PD2108-65, (*c*,*g*) PD2108-39, (*d*,*h*) PD2108-38. Scale bars: (*a*,*e*) 75 µm, (*b*,*f*) 70 µm, (*c*,*g*) 80 µm, (*d*,*h*) 85 µm.

We obtained 176 specimens that are unambiguously classified as *Spiralicellula*; 132 of the 176 specimens characterized tomographically were complete, and the remaining 44 specimens were incomplete owing to loss of cells or damage before or after fossilization (electronic supplementary material, S1). The complete fossils range in diameter from 462 to 1343 µm (median = 630.5 µm,), with an average of 667.47 µm (s.d. = 172.42, *n* = 132; figure 2). Few specimens preserve an outer envelope but this is likely an artefact since the diagnostic spiral structures on the component cells cannot be observed in specimens that preserve a complete outer envelope. Nevertheless, 58 specimens preserve an incomplete outer envelope and these occur across the full range of variation in the number of component cells (figure 3). This demonstrates that an outer envelope was present across all known developmental stages. In all instances, the envelopes are thin (less than 2 µm), single-layered and smooth or ornamented, showing evidence of both plastic (pre-fossilization) and brittle (post-fossilization) deformation (figure 3). Envelope ornamentation is either botryoidal or polygonal (figure 3*a–d*).

**Figure 4.**
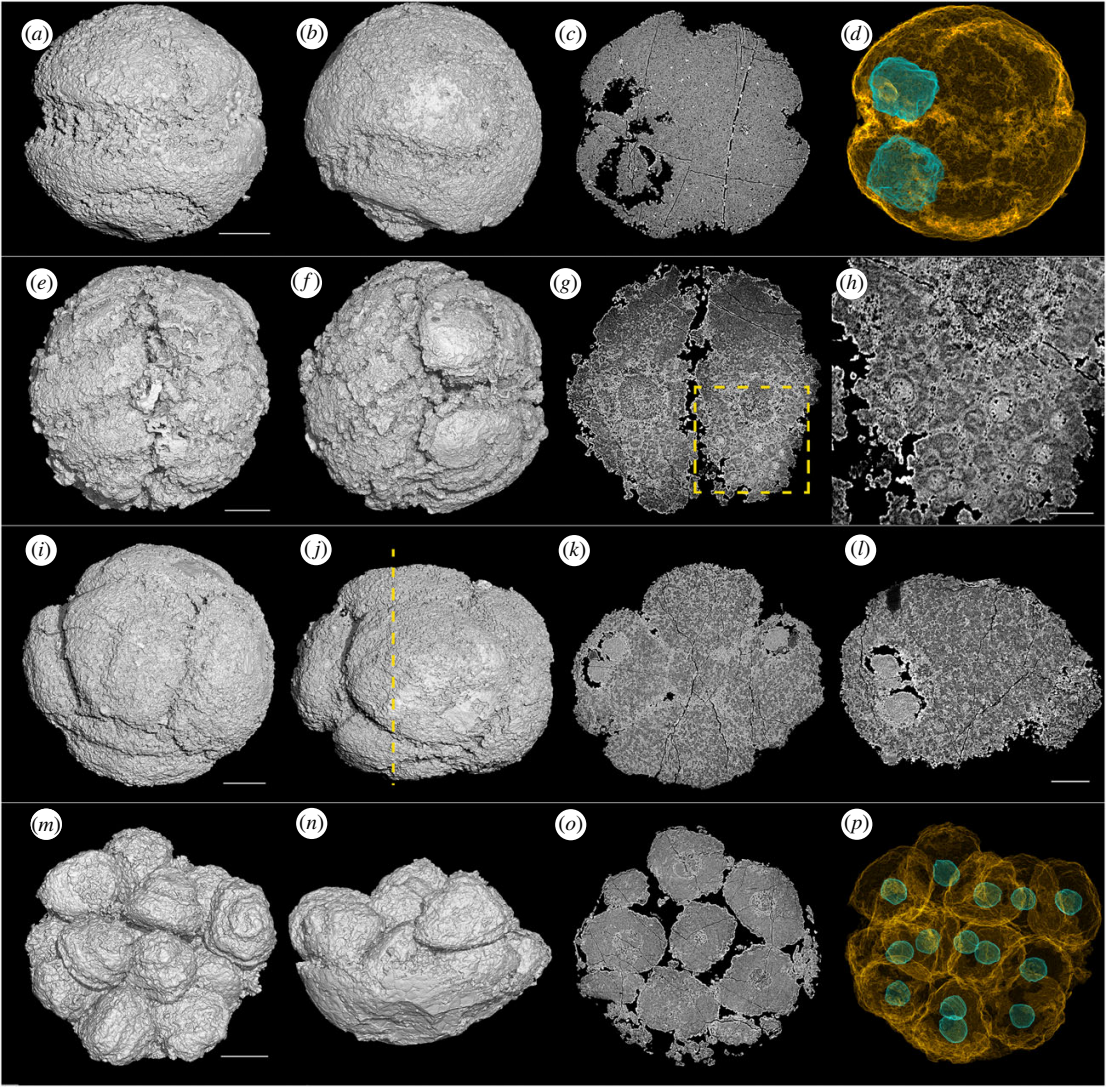
Cells and subcellular structures of *Spiralicellula.* (*a–d*) A 2-cell stage specimen. (*e–h*) A 4-cell stage specimen. (*i–l*) A 7-cell stage specimen with two nuclei in one cell. (*m–p*) A broken specimen with 14 cells left. (*a*,*b*,*e*,*f*,*i*,*j*,*m*,*n*) Synchrotron radiation X-ray tomographic microscopy (srXTM) surface renderings showing the spiral structures on the surface. (*c*,*g*,*k*,*o*) Virtual sections showing the subcellular structures. (*h*) Close-up view of boxed area in (*g*). Dashed line in (*j*) represents the plane of section shown in (*k*). (*l*) Virtual section of the cell with two nuclei. (*d*,*p*) Transparent models showing the cell boundaries and nuclei. (*a–d*) PD2108-70, (*e–h*) PD2108-83, (*i–l*) PD2108-31, (*m–p*) PD2108-30. Scale bars: (*a–d*) 100 µm, (*e–g*) 100 µm, (*h*) 36 µm, (*i–k*) 120 µm, (*l*) 75 µm, (*m–p*) 140 µm.

**Figure 5.**
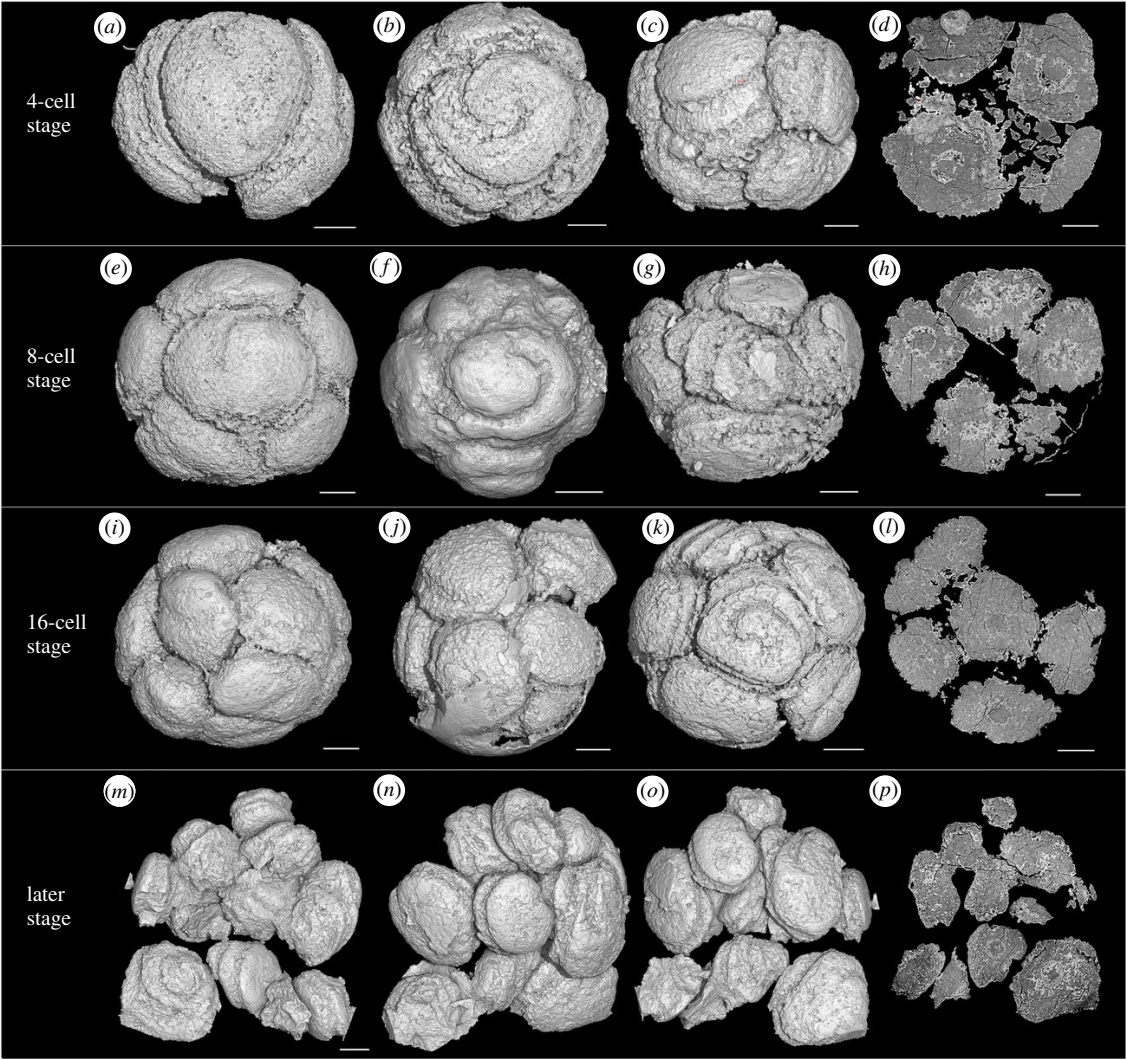
*Spiralicellula* at different developmental stages*.* (*a–c*,*e–g*,*i–k*,*m–o*) Synchrotron radiation X-ray tomographic microscopy (srXTM) surface renderings showing the spiral structures on the surface. (*d*,*h*,*l*,*p*) Virtual sections of (*c*,*g*,*k*,*o*) respectively. (*a*) PD2108-43, (*b*) PD2108-136, (*c*) PD2108-01, (*e*) PD2108-57, (*f*) PD2002-74, (*g*) PD2108-123, (*i*) PD2105-104, (*j*) PD2108-36, (*k*) PD2108-128. (*m*–*p*) PD2108-64. Scale bars: (*a*,*c*) 90 µm, (*b*,*h*) 95 µm, (*f*) 150 µm, (*m–p*) 85 µm, others 100 µm.

More than half of the specimens preserve two types of subcellular structure (figure 4), directly comparable to the interpreted nuclei (large spherical structures, around 50 µm in diameter; figure 4*c,d,k,l,o,p*) and yolk granules (small spherical structures, about 5–10 µm in diameter; figure 4*g,h*) in specimens of *Megasphaera* [9,36–38]. The large nuclei almost exclusively occur as one per cell (figure 4); one 7-cell stage specimen (figure 4*i–l*) includes one cell that is twice the volume of all others and preserves two nuclei within (figure 4*k,l*). The same asynchronous cell division phenomena can also be observed in *Megasphaera* [38]. Complete specimens contain 2, 4, 5, 7, 8, 9, 10, 12, 13 and 16 cells (electronic supplementary material, S1). In a 2-cell stage specimen, each cell is hemispherical and equal in volume (figure 4*a–d*). The 4- and 8-cell stage specimens account for the vast majority (figure 2). The total volumes of the component cells within specimens at different developmental stages stay consistently within a narrow and stable range. Furthermore, the diameters of these specimens remain unchanged regardless of the increase in the number of their inner cells. This is compatible with binary reductive palintomy observed in co-occurring embryo-like *Megasphaera* [7,8,35]. Incomplete specimens occur across this range of cell numbers, and estimating the original cell number of these incomplete specimens proves challenging (figures 3*g*, 4*m–p*, 5*k,l*,*m–p*). However, one incomplete approximately hemispherical specimen is composed of 14 cells (figure 4*m–p*) and so it can reasonably be interpreted to have been originally composed of about 28 cells.

The morphology and geometric relationships of the component cells vary among specimens. This is most evident in 4-cell specimens where (i) the cells are elongate and arranged in perpendicular pairs (figure 4*e–h*), (ii) the cells are elongate and arranged in parallel (figure 5*a*), or (iii) the cells are approximately equant and arranged in a tetrahedron (figure 5*b*). In many specimens, the cells are tightly packed, with little or no space between adjacent cells, whereas in others the cells are only loosely aggregated, with intervening voids (figure 5*c,g,j,m*). Late diagenetic cements and/or phosphatized bacterial filaments fill the intervening voids and so the cells are preserved in association, maintaining the integrity of complete specimens (figures 3*f*, 4*o* and 5*d,h,p*).

The spiral structures are not limited to the exposed surface of the cells; they continue onto the lateral surface between adjacent cells (figures 3*g,* 4*m,n* and 5*i,m–o*). The number of whorls of the spiral varies among specimens, and in 4- to16-cell specimens the spiral structure can range from one whorl on the outer surface of the cells, to three whorls that extend over the full surface of cells (figures 4*e,m* and 5*k*); there is no simple correlation between the number of cells and the degree of development of the surface spiral (figure 5). Nevertheless, the greater the development of the surface spiral, the looser the contact between component cells. The orientation of the cells can be inferred based on the axis of coiling of the surface spirals. In some specimens, the starting point of the spiral is visible on the outer surface of the specimen and the spiral axis of every cell is aligned radially with respect to the centre of the cell cluster (figures 3*g*,*h* and 5*b*,*e*,*f*,*k*). In other specimens, the spiral axis of cells is aligned at a tangent to the surface of the cell cluster (figures 4*e* and 5*c,g,j*). The spiral axis can also vary between cells in the same specimens (figures 4*m–p* and 5*m–p*).

## 4. Discussion

### (a) Developmental biology of *Spiralicellula*

While the majority of our specimens do not preserve an envelope, its fragmentary presence across different developmental stages—evident in specimens with varying cell numbers—suggests that an envelope was present in all known developmental stages. Thus, the absence or incomplete preservation of an envelope likely reflects taphonomy and not biology. Variation in cell size and number indicates that the cells underwent binary reductive cell division. The increase in cell numbers during the developmental sequence does not follow an exponential pattern of twofold growth, indicating that cell division was asynchronous between cell lineages. For example, the occurrence of a 7-cell specimen (figure 4*i–l*) with one large cell preserving two nuclei, twice the volume of the other six mononucleated cells, indicates that nuclear division preceded division of the cytoplasm and cell wall.

The degree of development of the spiral on the cell surfaces varies from just the surface of the cell facing the envelope, to development across the complete surface of the cell. This pattern of variation occurs across the range of palintomic stages, indicating that it does not correlate with overall development. Instead, we infer that the spiral develops anew at each palintomic stage, associated with the disaggregation of the cells. Indeed, the development of the spiral may be causal to the disaggregation of the cells. Orientation of the spiral axis provides a frame of reference for tracking the orientation of the component cells, demonstrating that they underwent significant and uncoordinated reorientation between rounds of palintomy. Together, cell disaggregation and uncoordinated reorientation suggest that cells behaved more like individuals than as coordinated components in the embryo of a multicellular adult.

Previous diagnoses of *S. bulbifera* limited the species to specimens composed of 4–16 cells; we extend this range from 2- to approximately 32-cell specimens (figure 3*a–d*,*m–p*). Obviously, we anticipate a single cell progenitor but it is not clear that we should expect to find specimens with more than 32 cells. It is possible that the spiral structure may be challenging to discern in specimens with a greater number of cells. However, these spiral structures do not become less distinguishable across the known palintomic stages. They would be easily identifiable in specimens at the 64- and 128-cell stages, where cells are half or a quarter the size of those at the 32-cell stage, respectively. This cell size relationship results from the palintomic cell division process, during which the cell count doubles, halving each cell's volume. Consequently, the volume of an individual cell is halved at the 64-cell stage and quartered at the 128-cell stage, compared with its volume at the 32-cell stage.

**Figure 6.**
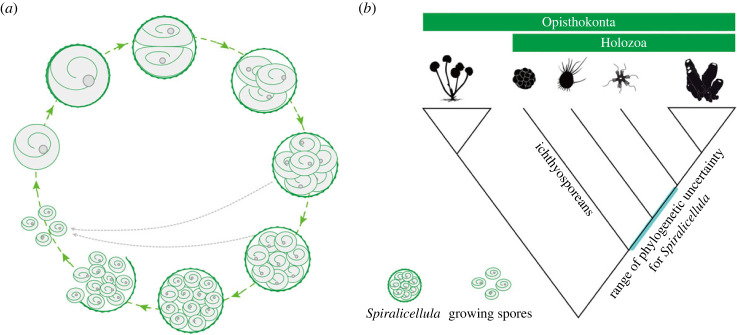
(*a*) Life cycle of *Spiralicellula.* Since earlier palintomic stages are also often incomplete and disaggregated, it is likely that there is no deterministic number of palintomic rounds, and cells are shed into the environment at earlier stages, which is shown as grey dashed arrows in the figure. (*b*) A simplified phylogenetic tree of Holozoa, with fungi as the outgroup. The potential placements for *Spiralicellula* in the holozoan tree are indicated in cyan.

It is possible that we have not encountered specimens with more than 32 cells because they might be more fragile and susceptible to damage during biostratinomic processes (cf. *Parapandorina*-stage *Megasphaera* in [39,40]). However, based on the available evidence we infer that the 32-cell stage approaches the upper limit for *Spiralicellula*. After all, our fossil collection is predominantly composed of specimens with low cell numbers, with the 8-cell stage specimens dominating the population. Specimens with a larger number of cells are among the most incomplete and poorly aggregated. We therefore conclude that the low number of palintomic stages reflects the end of this multicellular episode in the life cycle of *Spiralicellula*, after which the component cells separate and are released to the environment as individuals or gametes (figure 6*a*). Furthermore, since earlier palintomic stages are also often incomplete and disaggregated, it is likely that palintomy was not deterministic and cells were shed to the environment at earlier stages too (figure 6*a*). Thinning and disintegration of the envelope may, therefore, be biological, not (just) taphonomic.

### (b) The non-metazoan holozoan affinity of *Spiralicellula*

Previous attempts to resolve the affinity of *Spiralicellula* relied upon a developmental association with other embryo-like taxa in the Weng'an biota. But these links were weakly supported and were ultimately rejected [30]. Here, we attempt to explore the phylogenetic affinity of *Spiralicellula* based on its preserved morphology, anatomy and development biology. The well preserved nuclei in many specimens allow us to conclude that *Spiralicellula* is a eukaryote, at the very least. The multicellular stages resulting from palintomic cell division, exhibited by *Spiralicellula,* can be found in various lineages of eukaryote, including animal embryos, diverse clades of multicellular algae, and some non-metazoan holozoans.

We infer that *Spiralicellula* undergoes binary reductive palintomy within an enveloping cyst, yielding gametes or free-living cells [21,30]. This is in contrast to embryonic development in the multifarious clades of multicellular algae, where a mucilaginous envelope develops after the initiation of palintomy but does not proceed beyond a few rounds before undergoing cell differentiation [30,41]. Volvocalean algae undergo a much greater number of rounds of palintomy, retaining coherence of the embryonic cell cluster through incomplete cytoplasmic division [42,43]. However, this is clearly not the case in *Spiralicellula* or any other embryo-like fossils of the Weng'an biota, as evidenced by cell rearrangement and reorientation during development [41].

The embryo-like fossils in the Weng'an biota were once thought to represent the earliest metazoans based on the binary reductive palintomy of cells enclosed within an ornamented cyst wall [7,31]. But this is a widespread phenomenon among clonal multicellular eukaryotes [44]. More importantly*, Spiralicellula* does not exhibit characteristics of complex development, such as cell apoptosis and rearrangement, nor does it exhibit cell or tissue differentiation as part of a broader life cycle (table 1). Instead, the cell disaggregation and uncoordinated cell reorientation exhibited by *Spiralicellula* suggest that the cells behaved more like individuals than as coordinated components of a multicellular embryo. Consequently, we can reject a crown-group metazoan affinity for *Spiralicellula* (table 1).

**Table 1.** Comparison of characters among *Spiralicellula,* co-occurring Weng'an fossils and their analogues ‘○’ means presence of this character; ‘/’ means absence of this character; ‘?’ means unknown.

	spherical morphology	ornamented cyst	palintomy cleavage	multicellularity	Y-shaped junctions between cells	cell differentiation	tissue differentiation
*Spiralicellula*	○	○	○	○	○	/	/
*Megasphaera*	○	○	○	○	○	?	/
*Helicoforamina*	○	○	○	○	○	/	/
ichthyosporeans	○	○	○	○	○	/	/
metazoan embryos	○	○	○	○	○	○	○
stem metazoans	?	?	○	○	○	○	?

In terms of morphology, developmental mode, cell geometry and envelope histology, *Spiralicellula* exhibits considerable similarities to ichthyosporeans among non-metazoan holozoans (table 1). Ichthyosporeans develop endospores (daughter cells) inside a cyst (the cell wall of the mother cell) during their life cycle [45,46]. The development and reproduction of ichthyosporeans is compatible with the observed biological features and life cycle of *Spiralicellula.* Given the absence of evidence of cell differentiation during development in both *Spiralicellula* and ichthyosporeans (table 1), we posit that *Spiralicellula* is more likely affiliated with non-metazoan holozoans than with stem metazoans, which would be expected to exhibit at least some cell differentiation [22].

### (c) Implications for the timing of animal diversification

While the embryo-like fossils from the Weng'an biota were once thought to represent the earliest metazoans, recent research has suggested that some species, including *Caveasphaera* [40], *Helicoforamina* [29], *Ostiosphaera* [47], *Sporosphaera* [33] and now *Spiralicellula*, have affinities that lie outside crown Metazoa. Interpretations of soma–germ cell differentiation in *Megasphaera* [21] have been used to support their interpretation as stem metazoans, at best. The primary evidence for a stem-metazoan affinity of *Megasphaera* derives from ‘matryoshka’ structures that are interpreted to reflect cell differentiation. Nevertheless, it remains unclear whether these structures are endogenous or exogenous in origin [7,48].

The pattern of development seen in *Spiralicellula* is not readily distinguishable from the early stages of development described in *Megasphaera* [9,21,38]. *Spiralicellula* and *Megasphaera* possess a similarly structured and ornamented envelope (where it is fully preserved). They are comparable in size, and exhibit a similar nucleus/cytoplasm volume ratio across the first few rounds of palintomy [38]. The principal differences are the spiral cells and limited rounds of palintomy exhibited by *Spiralicellula*; the cells of *Megasphaera* exhibit many more rounds of palintomy. This is evidenced by the thousands of cells that compose ‘*Megaclonophycus*’-stage specimens attributed to *Megasphaera* [8,9,22,31,49]. Therefore, the similarities shared by *Spiralicellula* and *Megasphaera*, combined with the inferred non-metazoan holozoan affinity of *Spiralicellula*, force us to consider whether the affinity of *Megasphaera* also lies deeper within Holozoa. It is essential to note that the precise placement of *Megasphaera* within the Holozoa tree remains an open question. The possibility that the ‘matryoshka’ structures of *Megasphaera* are endogenous cannot yet be completely ruled out. Resolving this controversy definitively will require future efforts in three-dimensional reconstruction of a large number of *Megasphaera* specimens to fully elucidate its developmental processes and life cycle. This will help determine whether the ‘matryoshka’ structures are parasitic or a feature of cellular differentiation during development. Beyond these scenarios, another potential but yet unconfirmed explanation for the emergence of ‘matryoshka’ structures could involve asynchronous palintomic cell division. That is, a cluster of smaller cells enveloped by many larger cells could form if some cells divide significantly faster than others, leading to the characteristic ‘matryoshka’ structure.

The Weng'an biota, renowned for its exceptional preservation fidelity, is considered a distinctive taphonomic window that holds great potential for documenting the earliest metazoans. The absence of definitive evidence of crown metazoans in the biota is inconsistent with the expectations of the molecular clock estimates which posit a Tonian or Cryogenian origin for the clade [1,2]. It remains formally possible that the absence of crown-group animals from the Weng'an biota and earlier strata reflects the incompleteness of the fossil record, and the discovery of unequivocal metazoans from the Weng'an biota or older strata remains a viable possibility, not least given the discovery of crown metazoans, including cnidarians and bilaterians, within the later Ediacaran [50–54]. However, claims of crown metazoans from the Cryogenian [55,56] and Tonian [57,58] are all highly contested [59–62] and intense exploration of the Weng'an biota, the most exceptional of all sites of fossil preservation, has failed to yield the anticipated evidence of early crown metazoans, instead yielding only evidence of non-metazoan holozoans or possible stem metazoans. Alongside the Weng'an biota, the Doushantuo silicified Lagerstätte in South China serves as its lateral counterpart [28,63–65]. Despite differing preservational settings, this Lagerstätte remarkably preserves fossil structures down to a subcellular level. It contains a diverse array of microfossils, including cyanobacteria, acritarchs, multicellular algae, and embryo-like fossils [63,66,67], all found in the Weng'an biota. Notably absent, however, are fossils of crown-group metazoans. As such, the available fossil evidence suggests a relatively low probability of crown metazoans diversifying in the early Ediacaran, rather than ecological constraints within the Weng'an biota's preservational setting. Such insights prompt a recalibration of molecular timescales in light of these discoveries.

## Data Availability

All the specimens cited in this paper have been deposited in NIGPAS and the tomographic data arising from our study are available from the NIGPAS database at https://doi.org/10.12091/fossil-ontology.20240104 [68]. Supplementary material is available online [69].
